# The Impact of Child Dental Caries and the Associated Factors on Child and Family Quality of Life

**DOI:** 10.1155/2023/4335796

**Published:** 2023-07-29

**Authors:** Marziyeh Shokravi, Fatemeh Khani-Varzgan, Mohammad Asghari-Jafarabadi, Leila Erfanparast, Behjat Shokrvash

**Affiliations:** ^1^Department of Pediatric Dentistry, Faculty of Dentistry, Qazvin University of Medical Science, Qazvin, Iran; ^2^Dental caries Prevention Research Center, Qazvin University Of Medical Science, Qazvin, Iran; ^3^Department of Health Education and Promotion, Faculty of Health, Tabriz University of Medical Sciences, Golgasht Street, Tabriz, Iran; ^4^Cabrini Research, Cabrini Health, Melbourne, VIC 3144, Australia; ^5^Biostatistics Unit, School of Public Health and Preventative Medicine, Faculty of Medicine, Nursing and Health Sciences, Monash University, Melbourne, VIC 3004, Australia; ^6^Department of Psychiatry, School of Clinical Sciences, Faculty of Medicine, Nursing and Health Sciences, Monash University, Clayton, VIC 3168, Australia; ^7^Road Traffic Injury Research Center, Tabriz University of Medical Sciences, Tabriz, Iran; ^8^Department of Pediatric Dentistry, Faculty of Dentistry, Tabriz University of Medical Sciences, Golgasht Street, Tabriz, Iran; ^9^Medical Education Research Center, Health Management and Safety Promotion Research Institute, Tabriz University of Medical Sciences, Tabriz, Iran

## Abstract

**Introduction:**

Dental caries are considered as common health hazards and a serious lifelong threat to general health and quality of life. The present study aimed at identifying the impact of child dental caries and the associated factors on both child and family quality of life (QoL). *Material and Methods*. In this cross-sectional study, preschool children were selected randomly through clustered sampling from five educational districts in Tabriz, Iran, 2016. To assess the QoL related to oral health, the Early Childhood Oral Health Impact Scale (ECOHIS) was used. Clinical oral examination was performed to assess the presence of caries through the index of decayed, missing, and filled teeth (dmft). Descriptive and analytic statistical methods were used. To assess the underlying predictors of ECOHIS as a whole scale and the dimensions as a linear regression model were used as univariate and multivariate.

**Results:**

: Out of 756 children under 7 years old, 51.5% boys, mean (M) standard deviation (SD) of age 5.76 (0.78). About 85% of children had dental caries. The predictors of suboptimal ECOHIS related to both general and child dimensions were child age 6 year and above: *r* (756) = 2.43, and *P* ≤ 0.001, low-socioeconomic status: *r* (756) = 3.36 and *P* < 0.001 and high dmft: *r* (756) = 9.10 and *P* < 0.001. The predictors of suboptimal ECOHIS related to family domain were sex (girl): *r* (756) = 0.39 and *P* = 0.047; mother education (under12): *r* (756) = −0.92 and *P* < 0.001; mother job (employed) as univariate: *r* (756) = 0.71 and *P* = 0.002); and dmft: *r* (756) = −0.58 and *P* = 0.035.

**Conclusion:**

Adverse oral health of children imposes adverse effects on the QoL of children and families. Children's age, family socioeconomic level, presence of dental caries, child's gender, and mother's educational level were associated with the impact on QoL.

## 1. Introduction

Comprehensive health can be evaluated by indices of well-being and quality of life (QoL) in general, and oral health in particular [1, 2]. Dental caries are still considered as common health hazards and a serious lifelong threat to the general health and QoL. The global prevalence of dental caries appears in different patterns [3, 4]. While the unhealthy oral patterns have been decreasing in developed countries, they have shown an increasing process in developing societies, resulting in hours and hours of useful labor go to waste [3]. The total index of the Disability Adjusted Life Years indicates an increase of 20.8% because of unhealthy oral conditions between the years of 1990–2010. The World Health Organization (WHO) global strategy for prevention and control of noncommunicable diseases and the “common risk factor approach” offer new ways of managing the prevention and control of oral diseases [5]. The organization has also introduced the QoL as a significant part of oral health status and a supplementary part of school health management to achieve the optimum health of children [2, 3, 6].

Oral health-related quality of life (OHRQoL) is considered as an appropriate tool in assessing needs as well as implementing oral services in individual and public levels [2]. An increasing interest is observed in using Early Childhood Oral Health Impact Scale (ECOHIS) in the literature [2, 7]. It is related to the child characteristics [8, 9]. on the other hand, it also deals with family and environmental issues [10–12]. The relationship between parental features [13, 14], and the diverse oral health and ECOHIS was studied among different populations. The focus was put on axial role of parents in understanding child's general health [8, 9, 15], and adverse physical, psychological, and social consequences imposed by unhealthy oral status of the child [9, 10]. The effects on the family [2, 16] and the life quality of the parents were also studied. In general, family socioeconomic status and behavioral factors including dental visit behaviors along with implementation of preventive methods since the teeth eruption were determined as significant factors [3, 16, 17]. These factors are measures that influence child oral health and well-being indices [3, 16].

Comparing with the children of other East Mediterranean Countries, Iranian children were generally of higher health indices, except for the oral health [18]. The evidence shows that the oral health status of children over 12 and adults in Iran was reported as being poor [19]. Child oral health status among families with higher literacy and economic conditions has been described as satisfactorily [20, 21]. In addition the significant relationship was found between parental, and child oral health behaviors, and also between dental caries and OHRQoL [22, 23]. In hence, oral health literacy, attitude, and oral health behaviors of parents were considered as the important factors on child oral health [22, 23].

At the global level, numerous studies on the impact of dental caries on child and family QoL have been conducted [3, 14–17, 23]. In these studies, tooth decay has been found to be a significant predictor of poor QoL of the child and the family. In addition, the relationship between parental and child health behaviors has also been measured [23]. The impact of oral health of preschool children and some related factors on QoL of child were also measured in Shiraz city of Iran [24]. The significant relationship between child oral behavior, mother attitude, and OHRQoL were detected. However, the evidence of the associated factors including social economic status on OHRQoL among Iranian preschool children was not sufficient [24]. Thus, the present study was specifically conducted among preschool children of Tabriz city to identify the impact of child dental caries and the associated factors of child and family's QoL. It is expected that the results would be useful in planning programs of childhood health promotion and preventive interventions.

## 2. Material and Methods

### 2.1. Study Design

This cross-sectional study performed in 2016 with participation of 756 preschool children and their parents. The study was reported according to the Strengthening the Reporting of Observational Studies in Epidemiology (STROBE) guidelines. The institutional review board (IRB) of Tabriz University of Medical Sciences confirmed the study and referred the researchers to the educational officials and authorities to select the kindergartens and preschoolers randomly. We utilized a two-stage cluster randomized sampling approach for our study. Initially, we randomly selected 20% (approximately 15) of the 75 public kindergartens across five districts of Tabriz city using a random sequence generated by MS Excel. This was achieved by running the function 15 times to produce 15 distinct random numbers between 1 and 75, excluding any repeated numbers. In the subsequent stage, we again employed the random sequence generated by MS Excel to randomly select children and their parents from each kindergarten. To do so, we ran the function *k* times to generate *k* unique random numbers between 1 and *p*, excluding any duplicates. Here, *k* denotes the number of samples allocated to a particular kindergarten, and *p* refers to the number of children (and therefore their parents) enrolled in that kindergarten. The required sample size was estimated as 384 cases; considering 95% confidence level, marginal error of 5%, and utilizing Morgan Table. Taking the design effect of two, the final sample size was increased to 768 participants. A total number of 756 entered the study, based on eligibility criteria (parental consent and healthy children). After choosing the kindergarten according to above mentioned procedure, each was allocated a number of sample consistent with the proportion to the size of the kindergarten. Twelve children were excluded from the study because of absence, lack of child cooperation in oral examination, not brushed teeth, and uncompleted questionnaire by the parents.

### 2.2. Instruments

A multisection questionnaire and oral examination were used to data collection. The questionnaire consisted of three parts of demographic data of children and the parents, Family Affluence Scale (FAS) [25], or a list of household belongings which was validated earlier in Iran [26] scoring from 0 to 3 and above. The total scoring was within 5–21. The score of 5–10 was grouped as low, 11–13 middle, and 14 and above as high class. The cronbach's *α* coefficient and intraclass correlation coefficient (ICC) value estimated 0.87, and 0.80 indicating good level of internal consistency and stability reliability.

The third part included a valid Persian version of F-ECOHIS questionnaire [24, 27]. The origin version of ECOHIS questionnaire developed by Pahel et al. [28], which consisted of 16 items in two separate sections: child and family ECOHIS domains. The first section contained the items about the effects of oral health on child, and the second section about effects on the family. The child domain of ECOHIS including the signs and symptoms consisted of (1 item), physical functioning (6 items), psychological functioning or sensation (2 items), and social interaction (2 items). The second section included parent distress and the impact on family interactions and economics (5 items altogether).

The questions from parents were: since the teeth eruption; how many times your child has experienced toothache related restlessness, anxiety, trouble in drinking, eating, talking, pronouncing words, smiling or laughing, social interacting, sleeping, and absence from school? In the family scope the questions were: how many times the child oral health problems affected the family to experience; guilt feelings, absence from work, trouble in housework, and economic pressure? A question that does not belong to the validated questionnaire was added: “In general how satisfied are you about the oral health condition of your child.?” The scoring was done following Likert method choices (never = 0, seldom = 1, sometimes = 2, several times = 3, and frequently = 4). The total score was obtained by summing up the item scores of the total ECOHIS and domains. The possible total score of ECOHIS was (0–65, child domain was 0–45, and family domain was 0–20. The highest score was indicative of the highest adverse effects or suboptimal and vice versa.

### 2.3. Data Collection

The questionnaires along with the consent form were sent in advance to the children's parents or caregiver to fill out and return on the designated day for children oral examination. The children oral health status was examined by a single trained examiner using the dmft index [5, 29]. All children examined one by one using a probe, mirror, and under natural light (unless a flashlight needed) in a kindergarten class after brushing their teeth, and the number of decayed, missing, and filled teeth were diagnosed and recorded in the corresponding form.

### 2.4. Data Analyses

Data were presented using M (SD), median (min–max) for numeric normal and nonnormal variables, and frequency (percent) was presented for nonnumerical variables. To compare the child sex and age group differences dmft index, nonparametric Mann–Whitney *U* test, and Kruskal–Wallis test were used, respectively. The independent sample *t* test was used to compare the ECOHIS differences based on child age, and child sex. To determine the correlation between mother perceptions of ECOHIS as a whole scale and a subscale with the FAS or socioeconomic status of the family Anova test was used. To assess the underlying predictors of ECOHIS as a whole scale, and also as a subscale, separately for both the child and the family domains, linear regression were used as univariate and multivariate analyses. On univariat regression analysis total scores of ECOHIS considered as a dependent variable and child sex, child age, mother education, mother job, FAS, and dmft as an independent variable entered the model. We carried out the invariable and multivariable general linear model, and the results of multivariate analyses shown in the Table 1.

Statistical analysis was done by STATA software version 13 (Stata Corp, College Station, Texas 77,845 USA), and *P*-value of less than 0.05 was considered significant. The parents who refused to respond and those items which were not responded were considered as missed data and were excluded. There was no missing data detected on the dmft and ECOHIS items.

## 3. Results

### 3.1. Sample Characteristics

Of all 756 preschool children, 51.5% were boys with the age group of 4–7, and M (SD) of age 5.76 (0.78). Majority of mothers (77.6%) were unemployed, and over half of the mothers (61.5%) less than 12 years (Table 2). The oral health of preschool children based on the median (25th–75th percentile) of dmft index in boys and girls was 4 (2–9) and 5 (2–8), respectively, and the difference was not significant (*P* = 0.675). Only 15.2% (95% CI = 12.6–18.0) of children were dental caries free (Table 2).

### 3.2. Distribution of the ECOHIS Scores According to Sample Characteristics

The M (SD) of ECOHIS score as a whole scale was 16.45 (10.01); score for the child domain 9.06 (8.51); score for the family domain 6.51 (2.56). Significant differences were shown on the total score of the ECOHIS (*P* = 0.001), and total score for the child domain (*P* = 0.01). In family domain no significant differences were noticed (*P* = 0.246). The M (SD) of total score for the family domain of ECOHIS score was significant only based on the mother job (*P* = 0.002). In other variables no significant differences were noticed (Table 3). The M (SD) of the ECOHIS scores in child domain was significant based on mother education levels just on psychological scope (*P* = 0.031). It is not shown in the table.

### 3.3. Predictors of Total ECOHIS, Child and Family Domains Scores

A linear regression model was used as invariable and multivariable to determine the associated variables of ECOHIS, child and family domains. The predictors of suboptimal ECOHIS as a whole was child age (6 year and above): *r* (756) = 2.43 and *P* < 0.001, FAS (low): *r* = 3.36 and *P* < 0.001, and high dmft: *r* = 9.10 and *P* < 0.001. Adjusting for all variables such as child age, child sex, mother characteristics, FAS, and dmft index, the analysis show child age (6 year): *r* = 2.43 and *P* = 0.001, FAS (low): *r* = 3.36 and *P* < 0.001, and dmft (one and above): *r* = 9.10 and *P* < 0.001 were associated with total of ECOHIS scores (Table 1).

The predictors of suboptimal ECOHIS related to the child domain as an unvariable was child sex (girl): *r* (756) = −0.65 and *P* = 0.95, child age (6 year and above): *r* = 3.05 and *P* = 0.002; mother education (12 years and above): *r* = 1.30 and *P* = 0.420; mother job (employed): *r* = 1.35 and *P* = 0.089; FAS (low): *r* = 3.22 and *P* = <0.001; dmft (one and above): *r* = 9.41 and *P* = <0.001. Adjusting for all variables such as child age, child sex, mother characteristics, FAS, and dmft index, result show only FAS (low): *r* (765) = 2.71 and *P* = 0.003, and dmft: *r* = 9.12 and *P* = <0.001 were significant association (Table 1).The predictors of suboptimal ECOHIS related to the family domain as an unvariable was sex (girl): *r* = 0.59 and *P* = 0.030, child age (6 years and above): *r* = 0.98 and *P* = 0.040, mother education (12 years and above): *r* = 0.65 and *P* < 0.001, dmft (one and above): *r* = −060 and *P* = <0.001, and multivariate analysis results was child sex (girl): *r* = 0.39 and *P* = 0.047; mother education (12 years and above): *r* = −0.92and *P* < 0.001; dmft (one and above): *r* = −0.58 and *P* = 0.035 (Table 1).

## 4. Discussion

Following the estimation of child oral health condition using dmft index, the present research studied ECOHIS as an index to estimate the effects of general oral health status of children on (1) QoL of child and family as a whole; (2) child and family QoL separately (independently); (3) the predictors of ECOHIS: (a) child, (b) family domain.

The results revealed the predictors of suboptimal ECOHIS in child domain: child age (6 years and above), the socioeconomic status of the family, and dmft; on family domain: sex (girl) and mother educational level (13 years and above) as multivariate and mother job (unemployed) and mother educational level (13 years and above), and dmft as unvariable.

The percent of dental decay free and the median of dmft index were 15.2 and 4 (2–8), respectively, among children under study. However, the dmft index is far from the WHO goals to achieve the optimal dmft index (≤3) among children [5, 29]. The severity of dental caries of preschool children was significant based on the child sex. On the other hand, children oral health status based on dmft index (the number of decayed, missing, and filled teeth and the ratio of caries free teeth) showed a significant relationship with the parent perceptions of ECOHIS. The comparison of oral health of Iranian preschool children, based on dmft index [5, 29] revealed an unpleasant condition among the Iranian children. The condition was defined as mild in comparison with the East Mediterranean countries and as poor compared with the 3–6 year old children in Tehran city [20]. The prevalence of cavities was also higher among Iranian children comparing with the children in the developing and industrial countries such as Pakistan [30], Turkey [31], and the United States of America [32]. The possible reasons for the differences can be related to the location and the time differences of the studies in Iran, and the racial differences in other countries. In countries with high degree of tooth decay; educational programs of oral hygiene, accessibility to inexpensive facilities of dental care along with efficient health insurance should be offered and implanted.

The socioeconomic status of family, along with child age one of the main predictors of suboptimal ECOHIS as a whole scale and the child domain. In other word, the Iranian parent perception of oral health of children is primarily linked to the economic burden, the adverse oral health imposes on the family life rather than the adverse psychological influences it has on the child. It seems the rate of losing teeth increases with child aging which leads to emergence of more cavities. The possible trauma, especially on the anterior teeth, also interferes with freely speaking and expressing ideas and feelings by the child which in turn induces a negative effect on his/her QoL. Yet, Iranian parents were either totally ignorant of psychological or social interactional problems created by unhealthy oral status on their children life, or they overlooked it.

On family domain, the results revealed that the child sex (girl), mother characteristics, and dmft index were the main factors of the suboptimal ECOHIS. The condition of the child oral health related to QoL showed a significant relationship with the mother's personal characteristics.

The perception of mother of the QoL of female child (girl) among low-socioeconomic families is significant, with dmft. The reasons can be related to the female health [33] on low-socioeconomic families [8, 34], and mother particular perception of, and behavior with female child. The mother may be either too sensitive about the child's problems which in turn makes the child complain for more and more attention [34–36], or overlooks the comprehensive needs of the child which can be dental or other physical, psychological, and emotional related needs.

Considering ECOHIS in family domain reveals unexpected and somehow astonishing (startling) results. In families that mother is educated the family's QoL is reported undesirable. Although, it is expected that the educated and employed mothers be more concerned about their children QoL and more readily spend time and money for the child oral health which results in desirable QoL. It seems that these parents are unable to afford the high expenses of dental care. Therefore, the results of studies [37–40] come to disagreement with our findings. Some possible reasons can be attributed to the high costs of the dental cares, and or to the inability of the well-educated families to pay for it. It seems that the educated families avoid the on time and suitable dental cares despite being aware of their child's tooth problems. In order to obtain real results, these implications should be considered on the future research.

## 5. Conclusion

Adverse oral health of children imposes adverse effects on the QoL of children and families. Child age, family socioeconomic level, presence of dental caries, child sex, and mother's educational level were associated with the impact of dental caries on the QoL.

Regardless of having dental caries (dmft) or not, the perception of mother about QoL of her female child (girl) among low-socioeconomic families is undesirable. The problem is in need of in-depth investigations on parent general attitude toward female children.

To promote child's QoL and his/her appropriate growth and development, it is recommended to launch programs to enhance parental awareness before eruption of the deciduous or primary teeth and to develop an appropriate oral health insurance. The adverse oral health of child along with the child aging imposes distressful consequences on the routine activities and economic (money making) functioning of parents which imposes heavy costs especially on the low-level economic families. Thus families with high level of education and yet low-socioeconomic status are strongly affected by the adverse oral health of their children.

## Figures and Tables

**Table 1:** Results of multivariate analysis of underlying predictors of ECOHIS and subscales.

	Total ECOHIS score			Total ECOHIS score for child domain			Total ECOHIS score for family domain		
	Coefficient	95% CI^a^	*P* ^b^	Coefficient	95% CI^a^	*P* ^c^	Coefficient	95% CI^a^	*P* ^d^
Child sex									
Boy	Ref^e^			Ref^e^			Ref^e^		
Girl	−0.82	−2.18 to 0.55	0.241	−0.95	−2.18 to 0.27	0.125	0.39	0.01 to 0.77	0.047
Child age (year)									
4	Ref^e^			Ref^e^			Ref^e^		
5	0.42	−2.50 to 3.35	0.777	0.18	−2.40 to 2.76	0.894	0.46	−0.36 to 1.27	0.270
6	2.43	−0.33 to 5.19	0.001	1.45	−0.99 to 3.89	0.244	0.75	−0.02 to 1.51	0.055
7	2.42	−0.74 to 5.58	0.133	1.64	−1.17 to 4.44	0.252	0.50	−0.39 to 1.38	0.271
Mothers education (year)									
13 and above	Ref^e^			Ref^e^			Ref^e^		
0–12	−1.48	−3.21 to 0.25	0.094	0.71	−0.79 to 2.22	0.352	−0.92	−1.41 to −0.43	<0.001
Mother job									
Unemployed	Ref^e^			Ref^e^			Ref^e^		
Employed	−0.14	−1.77 to 2.06	0.885	−0.13	−1.81 to 1.55	0.877	0.18	−0.36 to 0.72	0.521
FAS^f^ (item)									
High (14 and above)	Ref^e^			Ref^e^			Ref^e^		
Middle (11–13)	0.71	−1.11 to 2.52	0.444	0.52	−1.06 to 2.09	0.521	0.08	−0.42 to 0.58	0.759
Low (5–10)	3.36	1.35 to 5.38	0.001	2.71	0.94 to 4.48	0.003	0.46	−0.11 to 1.03	0.111
dmft^g^									
Zero/none	Ref^e^			Ref^e^			Ref^e^		
One and above	9.10	6.90 to 11.30	<0.001	9.12	7.24 to 10.99	<0.001	−0.58	−1.18 to 0.03	0.035

^a^95% confidence interval, ^b^*P*-value < 0.05; multivariate analysis by quintile regression, adjusted for child age, child sex, mother education, mother job, FAS, dmft, ^c^Referent group ^d^Mean (standard deviation), ^e^Ref: reference group, ^f^FAS: Family Affluence Scale, ^g^dmft: decayed, missing, filled teeth.

**Table 2:** Sample characteristics.

	All 756 (100)^a^	Boy 389 (51.45)^a^	Girl 367 (48.54)^a^	*P*
Child age (year)				0.975^b^
4	53 (7.0)^a^	27 (50.1)^a^	26 (49.1)^a^	
5	185 (24.5)^a^	95 (51.4)^a^	90 (48.6)^a^	
6	405 (53.6)^a^	209 (51.6)^a^	196 (48.4)^a^	
7	113 (14.9)^a^	58 (51.3)^a^	55 (48.7)^a^	
All	5.76 (0.78)^d^	5.76 (0.77)^d^	5.76 (0.79)^d^	<0.001^b^
Mother education (year)				0.650^c^
0–12	465 (61.5)	243 (62.4)	220 (60)	
13 and above	291 (38.5)	146 (37.6)	147 (40)	
M (SD)^d^	12.43 (3.56)	12.24 (3.64)	12.63 (3.45)	
Mother job				0.650^c^
Unemployed	587 (77.6)	307 (78.9)	280 (76.3)	
Employed at home	20 (2.6)	9 (2.3)	11 (3)	
Employed out of home	149 (19.7)	73 (18.8)	76 (20.7)	
FAS^e^ (item)				0.002^c^
Low (5–10)	255 (33.7)	148 (38)	107 (29.2)	
Middle (11–13)	336 (44.4)	149 (38.3)	187 (51)	
High (14 and above)	165 (21.8)	92 (23.7)	73 (19.9)	
dmft index^f^	4 (2–8)^f^	4 (2–9)^f^	5 (2–8)^f^	<0.001^b^
Dental decay free^a^	115 (15.2)^a^	67 (17.2)^a^	48 (13.1)^a^	

^a^Number (percentage), ^b^*P*-value < 0.05; independent sample *t* test, ^c^*P*-value < 0.05; Chi-square test, ^d^Mean (standard deviation), ^e^Family Affluence Scale, ^f^Median (25th–75th).

**Table 3:** Distribution of ECOHIS scores according to sample characteristics.

	Total ECOHIS score^a^	Total score for the child domain^a^	Total score for the family domain^a^
Sex			
Boy (388/756)^b^	16.78 (10.47)	9.38 (8.95)	6.34 (2.65)
Girl (367/756)^b^	16.10 (9.48)	8.73 (8.02)	6.70 (2.46)
*P*^c^	0.353^c^	0.327^c^	0.064^c^
Child age (year)			
4	13.32 (8.79)	6.67 (7.86)	6.01 (2.570)
5	14.63 (8.82)	7.74 (7.87)	6.56 (2.29)
6	17.37 (10.23)	9.72 (8.74)	6.64 (2.65)
7	17.59 (10.91)	10.16 (8.65)	6.21 (2.67)
All	16.45 (10.01)	9.06 (8.51)	6.51 (2.57)
*p*^d^	0.001	0.01	0.246
Mother education (year)			
Under 12 (464)^b^	16.94 (10.35)	5.39 (4.56)	2.41 (2.28)
13 and above (291)^b^	15.66 (9.39)	4.40 (4.25)	2.13 (2.18)
*P*^c^	0,350^c^	0.189^c^	0.778^c^
Mother job			
Unemployed	15.60 (9.76)	8.03 (8.61)	7.10 (2.51)
Employed	16.69 (10.07)	9.37 (8.46)	6.35 (2.56)
*P*^c^	0.555	0.957	0.002
FAS^e^			
Low (5–10)	18.70 (10.07)	11.10 (8.46)	6.49 (2.88)
Medium (11–13)	15.47 (9.26)	8.25 (7.95)	6.48 (2.48)
High (14 and above)	14.98 (8.86)	7.88 (7.71)	6.61 (2.30)
*P*^d^	0.001	<0.001	0.867
dmft^f^			
Zero	10.19 (6.73)	2.80 (5.50)	7.35 (1.99)
One and above	17.58 (1011)	10.33 (8.42)	6.35 (2.63)
*P*^c^	<0.001	<0.001	<0.001

^a^Mean (standard deviation), ^b^Number, ^c^*P*-value based on independent samples *t* test, ^d^*P*-value based on One-way Anova, ^e^FAS Family Affluence scale. ^f^dmft: decayed, missing, filled teeth.

## Data Availability

All the raw data sets that support the findings of this study are available from the corresponding author (BS) upon reasonable request.

## Additional Points

*Limitations*. An estimation of oral health impact on QoL of children was done indirectly asking only parents. It must be emphasized that it is a common indicator among preschoolers [25]. Another limitation of the study is related to the teacher perception, which was not taken and it is recommended to be taken into consideration in other studies. It must be emphasized that dmft index as an expert indicator does not show the health progress over time.
